# Vigorous Physical Activity Is Associated With Better Glycated Hemoglobin and Lower Fear of Hypoglycemia Scores in Youth With Type 1 Diabetes: A 2-Year Follow-Up Study

**DOI:** 10.3389/fphys.2020.548417

**Published:** 2020-10-23

**Authors:** Georges Jabbour

**Affiliations:** Sport Science Program, College of Arts and Sciences, Qatar University, Doha, Qatar

**Keywords:** children, adolescent, type 1 diabetes, vigorous physical activity, glycated hemoglobin, fear of hypoglycemia, follow-up

## Abstract

To correlate glycated hemoglobin (HbA1c) and fear of hypoglycemia scores with physical activity (PA) levels in children and adolescents with type 1 diabetes (T1D) over a period of 2 years. Twenty-eight children and 33 adolescents with T1D have been assessed for their PA profile. Personal and medical data for the patients were collected at baseline (visit 0: V0), 1 year later (V1), and 2 years later (V2). At baseline, children with T1D engaged in less moderate to vigorous PA (MVPA) (*p* < 0.01) per day than adolescents. These results did not differ across visits. On the contrary, adolescents spent fewer time in vigorous physical activity (VPA) (*p* < 0.01) than children did (*p* < 0.01). Fear of hypoglycemia scores correlated significantly with VPA levels (β = −0.41, *p* = 0.03; β = −0.44, *p* = 0.06; β = −0.61, *p* = 0.001). For HbA1c (%), significant correlations were reported with VPA levels (β = −0.54, *p* = 0.02; β = −0.47, *p* = 0.03; β = −0.62, *p* = 0.01) across visits. Body mass index percentile correlated with total screen time (β = 0.28, *p* = 0.02; β = 0.29, *p* = 0.01; β = 0.31, *p* = 0.04) and overall PA levels (β = −0.52, *p* = 0.02; β = −0.42, *p* = 0.03; β = −0.42, *p* = 0.01). Performing more vigorous PA a day is associated with better HbA1c with lower perceived fear of hypoglycemia among youth with T1D. Therefore, dedicating more time in VPA may be an appropriate advice for patients with T1D.

## Introduction

In the context of type 1 diabetes (T1D) management, physical activity (PA) practices are widely recommended as a major preventive factor of the deleterious effects of T1D (Colberg et al., 2016). Indeed, to obtain optimum benefits from PA engagement, the Centers for Disease Control and Prevention, the American Heart Association, and the American Diabetes Association (Colberg et al., 2016) recommended that children and adolescents with T1D engage in at least 60 min/day of moderate- to vigorous-intensity aerobic activity, including vigorous muscle-strengthening and bone-strengthening activities at least 3 days/week.

Regular exercise can improve health and well-being and can help individuals achieve their target fitness and glycemic goals, and higher levels of PA are associated with improved glycemic control and a decrease in cardiovascular risk factors in individuals with T1D (Carral et al., 2013; Bohn et al., 2015). Moreover, the addition of structured exercise may potentiate these benefits (Valerio et al., 2007; Trigona et al., 2010). For Trigona et al. (2010), youth with T1D must go beyond simply being active to obtain the most benefit and improve their physical fitness profile, in addition to increasing their engagement in PA.

Despite the known benefits of PA, the numerous physiological (e.g., hypoglycemia episodes) and psychological (e.g., fear of hypoglycemia) risks associated with T1D make it challenging to incorporate PA practices (Riddell et al., 2017). Consequently, people with T1D tend to be at least as inactive as the general population, with a large percentage of individuals not achieving the minimum amount of moderate to vigorous aerobic activity per week (Øverby et al., 2009; Trigona et al., 2010). Several barriers to PA practices can exist for a person with T1D, leading to effects on the level and the mode of PA participation. These barriers have been evaluated using the Barriers to Physical Activity in Type 1 Diabetes (BAPAD-1) scale (Dubé et al., 2006; Brazeau et al., 2008, 2012b), and fear of hypoglycemia was identified as the main barrier to PA practices in adults (Brazeau et al., 2008) as well as in youth (children and adolescents) (Jabbour et al., 2016). Recently, a cross-sectional study conducted in diabetes adults supports the assumption that PA may be lower in this population due to unique barriers (Keshawarz et al., 2018). Unfortunately, reporting potential barriers, particularly fear of hypoglycemia in relation to PA levels is limited to cross-sectional studies, and no data are available whether any changes in scores for fear of hypoglycemia and/or overall perceived barriers scores over time will affect PA levels. Such data are crucial to add more emphasis on the relationship between PA levels and perceived barriers, open new insight toward engagement in PA safely, and increase overall PA.

Within this perspective, the present 2-year follow-up study aimed to explore the association between perceived barriers, mainly the fear of hypoglycemia, and PA using a detailed and validated questionnaire among youth with T1D. Since glycemic control and body weight control are important in improving overall health in individuals with T1D (Carral et al., 2013; Bohn et al., 2015) and known to be reduced by PA practices (Carral et al., 2013; Bohn et al., 2015), they will be explored throughout.

## Materials and Methods

### Study Population

The participants of this project are young people (boys and girls) with T1D (diagnosed for at least 1 year) and aged between 5 and 17 years old. The study was conducted at the Pediatric Diabetic Clinic of the Dr. Georges-L.-Dumont (Vitalité Health Network), located in Dieppe, in which 61 children and adolescents with T1D were followed (from a total of ∼630 patients registered in all the affiliated pediatric clinics of the Vitalité Health Network). The ethics committee of the Vitalité Health Network approved the project, and all participants signed an informed consent form. This study complies with the principles laid down in the Declaration of Helsinki Recommendations. Of the 61 children and adolescents with T1D, all agreed to take part in the study, representing 100% of the Pediatric Diabetic Clinic of the Dr. Georges-L.-Dumont’s clientele. All completed a self-administered questionnaire directly at the clinic administered by kinesiology students at the University of Moncton.

For the purpose of the present work, these same children and adolescents were asked to answer the same questionnaire 1 year (visit 1; V1) and 2 years later (visit 2; V2). Data were collected consecutively during the same period of collection (between October and November). All participants met the inclusion criteria: age between 6 and 17, duration of diabetes longer than 1 year, and no other chronic diseases.

**FIGURE 1:**
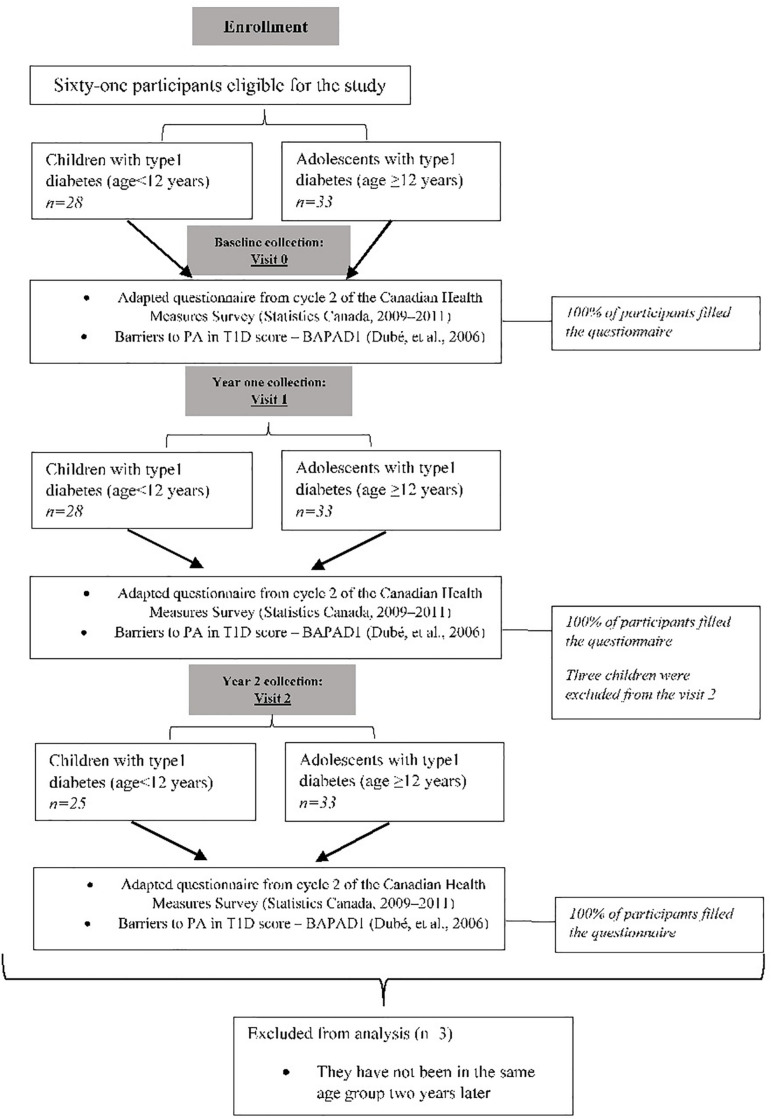
Flow chart of study design and participants’ enrollment.

In the present study (Figure 1), 61 participants completed all the relevant questionnaires at baseline [visit 0 (V0)] prior to their regular visit with the physician. They were asked to complete one questionnaire related to exercise barriers (barriers to PA in T1D score–BAPAD-1; for all, see Table 1) and the activity profile using the questionnaire from cycle 2 of the Canadian Health Measures Survey, which is adapted according to age (children and adolescents; < 12 years and ≥ 12 years old). More details are provided in the questionnaire section. All participants agreed to be contacted 1 and 2 years later for the same purpose [visits 1 and 2 (V1; V2)]. At both visits, all completed the same questionnaire that they completed at V0. However, three participants were excluded from the final analysis because they did not meet the age criteria (Figure 1). Ultimately, 58 participants separated into two groups (*n* = 25 children and *n* = 35 adolescents) were analyzed for the purposes of the present work.

**TABLE 1:** Descriptive characteristics and activity profile of children and adolescent with type 1 diabetes across visits.

	**Visit 0**		**Visit 1**		**Visit 2**		**Paired *t*-test**	
	**Children (*n* = 28)**	**Adolescents (*n* = 33)**	**Children (*n* = 28)**	**Adolescents (*n* = 33)**	**Children (*n* = 25)**	**Adolescents (*n* = 33)**	**FT**	**p**
**Study population characteristics**								
Sex (% boys)	55	57	55	57	60	57	–	–
Age (years)	7.1 ± 0.6	15.2 ± 0.3^*a*^	8.1 ± 0.7^*b*^	16.1 ± 0.6^*ab*^	9.2 ± 0.4^*b*^	17.3 ± 0.3^*ab*^	21	<0.01
Weight (kg)	30 ± 3	65 ± 3^*a*^	50 ± 2^*b*^	70 ± 10^*ab*^	59 ± 1^*b*^	74 ± 5^*ab*^	29	<0.01
Height (cm)	130 ± 3	165 ± 2^*a*^	136 ± 6^*b*^	164 ± 4^*ab*^	148 ± 4^*a*^	164 ± 8^*ab*^	32	<0.01
BMI percentile	61 ± 11	80 ± 13^*a*^	69 ± 17^*b*^	86 ± 14^*ab*^	72 ± 1 3^*b*^	88 ± 16^*ab*^	29	<0.01
HbA1c (%)	7.1 ± 1.9	7.2 ± 2.19	7.2 ± 0.6	7.3 ± 0.7	7.2 ± 1.1	7.1 ± 1.2	9.3	0.43
Sometimes experience PA hypoglycemia *(%)*	45 ± 3	76 ± 2^*a*^	40 ± 1	86 ± 2^*ab*^	46 ± 1	90 ± 2^*ab*^	19	<0.01
Sometimes experience PA hyperglycemia *(%)*	20 ± 3	21 ± 1	18 ± 2	18 ± 1	22 ± 1	17 ± 3	8.6	0.54
**Barriers to Active Lifestyles measured by the BAPAD-1**								
Diabetes-specific barriers to PA with score ≥ 4 n (%)	21	44	24	39	23	47		
Loss of control over diabetes	2.2 ± 1.6	2.8 ± 1.8	2.7 ± 1.6	3.6 ± 1.9	2.6 ± 1.1	3.2 ± 1.9	10.6	0.64
Fear of hypoglycemia	2.7 ± 1.4	3.1 ± 1.9	2.7 ± 1.6	2.8 ± 1.9	2.6 ± 1.9	3.2 ± 1.8^*ab*^	16	<0.05
Fact that you have diabetes	2.1 ± 1.5	2.4 ± 1.6	2.2 ± 1.5	2.9 ± 1.1	1.6 ± 1.7	3.1 ± 1.9^*a*^	14	<0.05
Risk of hyperglycemia	1.9 ± 1.1	2.1 ± 1.7	2.1 ± 1.1	1.9 ± 1.6	2.3 ± 1.4	1.9 ± 1.1	9.6	0.44
Diabetes-universal barriers to PA with score ≥ 4 n (%)	18	20	18	19	21	24		
Fear of hurting self	2.1 ± 1.1	1.9 ± 0.1	2.2 ± 1.2	1.8 ± 0.3	1.9 ± 0.8	1.8 ± 0.6	8.7	3.2
Low fitness level	2.1 ± 1.6	2.2 ± 0.9	2.1 ± 1.3	1.9 ± 0.7	2.1 ± 0.7	1.9 ± 0.9	7.7	2.4
Weather conditions	2.6 ± 1.6	2.6 ± 1.6^*a*^	2.7 ± 1.1	2.9 ± 1.4^*a*^	2.1 ± 1.7	3.2 ± 0.9^*a*^	14	<0.05
Sport Center proximity	1.8 ± 1.3	1.7 ± 1.1	1.6 ± 1.1	1.6 ± 1.1	1.7 ± 1.5	1.5 ± 0.7	9.7	4.4
School/Work schedule	1.6 ± 0.9	2.6 ± 1.2^*a*^	1.4 ± 0.7	2.9 ± 0.9^*a*^	1.1 ± 0.9	2.7 ± 1.2^*a*^	16	<0.05
Actual physical health status (excluding diabetes)	1.9 ± 1.5	1.8 ± 1.4	1.9 ± 0.9	1.9 ± 0.7	1.7 ± 0.5	1.7 ± 0.7	4.9	2.1
Total BAPAD-1 score	2.1 ± 1.4	2.3 ± 1.3	2.2 ± 0.9	2.4 ± 0.8	1.9 ± 0.8	2.4 ± 0.9	6.3	5.1
**Self-reported PA and screen time information**								
Total screen time⋅day^–1^ (h) (TV, video games, computer)	1.8 ± 1.2	2.8 ± 1.8^*a*^	1.6 ± 1.3	2.9 ± 1.1^*a*^	2.2 ± 1.2	2.9 ± 0.8^*a*^	26	<0.01
Total time in MVPA.day^–1^ (min)	41 ± 11	52 ± 14^*a*^	44 ± 09	56 ± 12^*a*^	41 ± 06	56 ± 13^*a*^	26	<0.01
Total time in VPA.day^–1^ (min)	27 ± 09	8 ± 02^*a*^	23 ± 07	5 ± 04^*a*^	26 ± 11	4.3 ± 03^*a*^	19	<0.01
Number of day spent on VPA per week	3.1 ± 0.2	1.8 ± 0.2^*a*^	2.9 ± 0.8	1.1 ± 0.3^*a*^	3.2 ± 0.8	1.4 ± 0.8^*a*^	15	<0.01

*Values are mean (standard deviation). BMI, body mass index; HbA1c, glycated hemoglobin; PA, physical activity; n (%), number in percentage; MVPA, moderate to vigorous physical activity; VPA, vigorous physical activity; BAPAD-1, Barriers to Physical Activity in Type 1 Diabetes; h, hour; min, minute. Significant difference between groups (^*a*^p < 0.01). Significant difference from previous visit values (^*b*^p < 0.01).*

Age and sex were obtained using a self-reported questionnaire completed by the child/adolescent. Height and weight were evaluated on-site by a nurse. Age- and sex-specific body mass index (BMI) percentiles were calculated according to the US Centers for Disease Control and Prevention growth charts (Lau, 2007; Centers for Diseases Control and Prevention, 2014). The number of years since the patient’s diabetes diagnosis was also calculated, and mean glycated hemoglobin (HbA1c) values over the preceding 3 months were preliminarily recorded.

### Barriers to Physical Activity

We administered the validated BAPAD-1 questionnaire to all study participants. The questionnaire consists of eight universal barriers to PA relevant to all study participants and four diabetes-specific barriers. For this study on children and adolescents, nine items were kept, since two items were not applicable to this age group (“the fear of suffering a heart attack” and “the fear of being tired”) and “the school schedule” was added (Jabbour et al., 2016; Michaud et al., 2017). The BAPAD-1 score was obtained by calculating the average of the individual scores obtained for each type of barrier, for which the answers to exercise barriers were rated from 1 (extremely improbable) to 7 (extremely probable). For the purposes of the present analysis, scores from 1 to 4 were categorized as “barrier not present,” while scores from 5 to 7 were categorized as “barrier present”.

### Activity and Sedentary Profile

To obtain the activity profile, we used the questionnaire from cycle 2 of the Canadian Health Measures Survey (Statistics Canada, 2009–2011). This questionnaire was developed and administered in two versions: one version for children under 12 and another version for adolescents 12 years and older. In this questionnaire, children and adolescents reported how many hours per day they usually spend engaged in sedentary activities, such as using a computer, playing video games, or watching TV/videos. For the data analysis, the three categories “none,” “<1 h/day,” and “1–2 h/day” were recoded as “≤2 h/day,” and the other categories (“3–4 h/day,” “5–6 h/day,” and “≥7 h/day”) were recoded as “>2 h/day,” which is the closest possible threshold within these categories to the cutoff, according to the Canadian guidelines for screen time (<2 h/day) (Canadian Society of Exercise Physiology, 2012).

Next, the average minutes per day spent in various forms of PA were derived. Using the World Health Organization norms on metabolic equivalents of tasks (METs) (World Health Organization, 2015) and Ainsworth’s Compendium of Physical Activities for children (Ridley et al., 2008), activity was categorized as low (≤3 METs), moderate (<3 METs ≤ 6), or vigorous (>6 METs) in intensity. This will allow us to calculate the amount of time spent daily in each PA intensity and to identify adolescents who follow the recommendations of ≥60 min of daily of moderate to vigorous PA (MVPA) (Ridley et al., 2008; McArdle et al., 2010).

### Statistical Analysis

The analyses were performed using the IBM SPSS v. 21 software (IBM, Armonk, New York, United States). The data are presented as means (standard deviations). Normality was tested using the Kolmogorov–Smirnov test. Paired *t*-tests were used to determine whether significant differences occurred at each visit within and between groups. We used multiple linear regression to model the mean outcomes for each exposure of interest. For both linear and logistic regressions, the independent variables considered in the regression models were BAPAD-1 score, perceived barrier items, and previous experiences of PA hypo/hyperglycemia. A value of *p* < 0.05 was set as the level of statistical significance.

## Results

The characteristics of the study participants were compared (Table 1). Among the children, 55% of the subjects were boys, except for visit 2, in which 60% of the subjects were boys (three children were excluded from the final analysis, see Figure 1). For the adolescent group, 60% of the subjects were boys (Table 1). Anthropometric variables (height, weight, BMI percentile) increased for both groups across visits (Table 1) and were significantly higher for adolescents than for children (*p* < 0.01, respectively). There were no significant differences in HbA1c (%) between groups across visits (Table 1). At visit 0, 45% of children reported sometimes experiencing hypoglycemia during PA, while this figure was 40% at visit 1 and 46% at visit 2. Among adolescents, 76% reported sometimes experiencing hypoglycemia during PA at visit 0, and this percentage increased significantly at visit 1 (86%; *p* < 0.01) and at visit 2 (90%; *p* < 0.01). The total scores for PA barriers monitored by the BAPAD-1 questionnaire were similar for both groups (Table 1) and across visits. However, the frequency with which adolescents assigned a score of 4 or greater to diabetes-specific barriers was two times greater than that reported by children (Table 1). The most common diabetes-specific barrier identified was the fear of hypoglycemia. This barrier increased among adolescents at V2 compared to those at V1 (*p* < 0.01).

**FIGURE 2:**
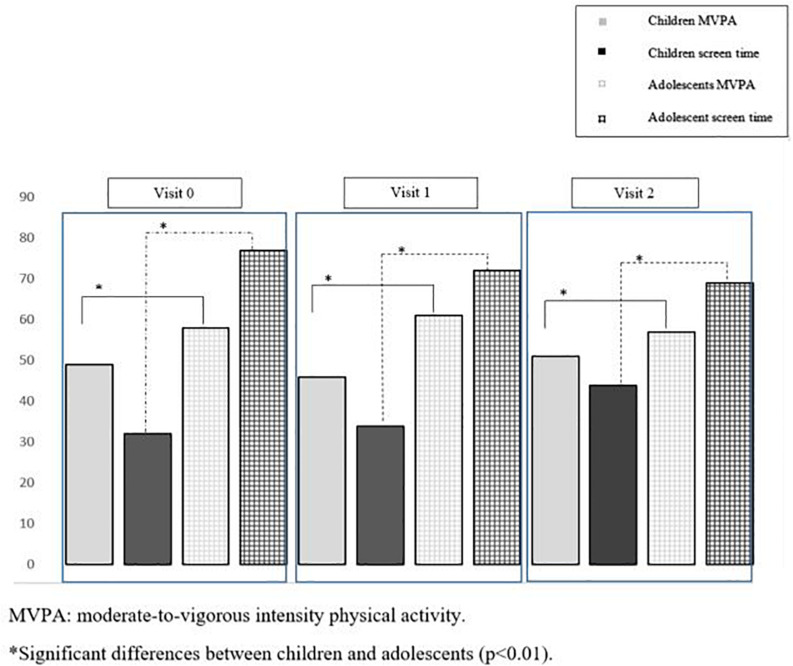
Percentage of children and adolescents above 60 min a day of moderate-to-vigorous intensity physical activity and above 2 h of screen time across visits.

At baseline, children engaged in less MVPA (*p* < 0.01) per day and had less screen activity (*p* < 0.01) than adolescents did. The frequency with which participants engaged in ≥ 60 min a day of moderate- to vigorous-intensity PA and ≥ 2 h of screen time across visits is presented in Figure 2. Approximately 30% of children vs. 80% of adolescents have at least 2 h of screen time per day. In contrast, adolescents spent less time in VPA (*p* < 0.01) and less time in VPA bouts per day (*p* < 0.01) than children did (Table 1).

To examine whether participants with T1D who reported diabetes-specific barriers had low levels of PA, we present results for the linear regression analysis of PA outcomes in Table 2. Participants with higher scores for barriers spent significantly less time in MVPA per day than participants with lower reported barriers; this pattern was seen across all visits (β = -0.12, *p* = 0.05; β = -0.14, *p* = 0.04; and β = -0.12, *p* = 0.01 for V0, V1, and V2, respectively). Moreover, VPA is significantly correlated with total barrier scores (Table 2). A higher “fear of hypoglycemia” score across visits was observed for those with less VPA (β = −0.41, *p* = 0.03; β = −0.44, *p* = 0.06; and β = −0.61, *p* = 0.001 for V0, V1, and V2, respectively).

**TABLE 2:** Standardized regression summary for total screen time, moderate to vigorous physical activity, and vigorous physical activity across the three visits.

	**Total screen time.day^–1^ (h)**			**Total MVPA.day**^–^**^1^ (min)**			**Total VPA.day**^–^**^1^ (min)**		
	**Visit 0**	**Visit 1**	**Visit 2**	**Visit 0**	**Visit 1**	**Visit 2**	**Visit 0**	**Visit 1**	**Visit 2**
BMI percentile	R^2^ adj. = 0.07; β = 0.28; *p* = 0.02*	R^2^ adj. = 0.08; β = 0.29; *p* = 0.01*	R^2^ adj. = 0.065; β = 0.31; *p* = 0.04*	R^2^ adj. = 0.039; β = −0.52; *p* = 0.02*	R^2^ adj. = 0.066; β = −0.42; *p* = 0.03*	R^2^ adj. = 0.054; β = −0.42; *p* = 0.01*	R^2^ adj. = 0.81; β = 0.03; *p* = 0.98	R^2^ adj. = 0.66; β = 0.06; *p* = 0.72	R^2^ adj. = 0.718; β = 0.05; *p* = 0.92
HbA1c (%)	R^2^adj. = 0.51; β = 0.04; *p* = 0.42	R^2^ adj. = 0.66; β = 0.04; *p* = 0.52	R^2^ adj. = 0.51; β = 0.03; *p* = 0.42	R^2^adj. = 0.67; β = 0.08; *p* = 0.72	R^2^ adj. = 0.46; β = 0.02; *p* = 0.32	R^2^ adj. = 0.71; β = 0.06; *p* = 0.62	R^2^ adj. = 0.044; β = −0.54; *p* = 0.02*	R^2^ adj. = 0.035; β = −0.47; *p* = 0.03*	R^2^ adj. = 0.063; β = −0.62; *p* = 0.01*
Sometimes experience PA hypoglycemia *(%)*	R^2^ adj. = 0.71; β = 0.10; *p* = 0.92	R^2^ adj. = 0.63; β = 0.04; *p* = 0.42	R^2^ adj. = 0.81; β = 0.09; *p* = 0.45	R^2^ adj. = 0.06; β = 0.61; *p* = 0.001*	R^2^ adj. = 0.05; β = 0.39; *p* = 0.001*	R^2^ adj. = 0.04; β = 0.21; *p* = 0.03*	R^2^ adj. = 0.71; β = 0.05; *p* = 0.58	R^2^ adj. = 0.62; β = 0.07; *p* = 0.64	R^2^ adj. = 0.73; β = 0.06; *p* = 0.63
Sometimes experience PA hyperglycemia *(%)*	R^2^ adj. = 0.05; β = 0.21; *p* = 0.04*	R^2^ = 0.04; β = 0.39; *p* = 0.03*	R^2^ adj. = 0.09; β = 0.51; *p* = 0.01*	R^2^ adj. = 0.04; β = 0.41; *p* = 0.003*	R^2^ adj. = 0.03; β = 0.33; *p* = 0.06*	R^2^ adj. = 0.06; β = 0.31; *p* = 0.04*	R^2^ adj. = 0.66; β = 0.07; *p* = 0.68	R^2^ adj. = 0.41; β = 0.04; *p* = 0.54	R^2^ adj. = 0.67; β = 0.04; *p* = 0.62
Loss of control over diabetes	R^2^ adj. = 0.61; β = 0.05; *p* = 0.62	R^2^ = 0.66; β = 0.07; *p* = 0.68	R^2^ adj. = 0.72; β = 0.05; *p* = 0.62	R^2^ adj. = 0.41; β = 0.04; *p* = 0.56	R^2^ adj. = 0.43; β = 0.06; *p* = 0.64	R^2^ adj. = 0.58; β = 0.03; *p* = 0.52	R^2^ adj. = 0.36; β = 0.03; *p* = 0.38	R^2^ adj. = 0.51; β = 0.06; *p* = 0.64	R^2^ adj. = 0.47; β = 0.06; *p* = 0.42
Fear of hypoglycemia	R^2^ adj. = 0.77; β = 0.04; *p* = 0.72	R^2^ = 0.76; β = 0.05; *p* = 0.65	R^2^ adj. = 0.62; β = 0.06; *p* = 0.42	R^2^ adj. = 0.61; β = 0.06; *p* = 0.68	R^2^ adj. = 0.67; β = 0.05; *p* = 0.61	R^2^ adj. = 0.63; β = 0.04; *p* = 0.53	R^2^ adj. = 0.05; β = −0.41; *p* = 0.03*	R^2^ adj. = 0.07; β = −0.44; *p* = 0.06*	R^2^ adj. = 0.08; β = −0.61; *p* = 0.001*
Fact that you have diabetes	R^2^ adj. = 0.46; β = 0.05; *p* = 0.42	R^2^ = 0.39; β = 0.06; *p* = 0.58	R^2^ adj. = 0.52; β = 0.06; *p* = 0.52	R^2^ adj. = 0.45; β = 0.03; *p* = 0.38	R^2^ adj. = 0.43; β = 0.03; *p* = 0.44	R^2^ adj. = 0.47; β = 0.03; *p* = 0.52	R^2^ adj. = 0.26; β = 0.02; *p* = 0.32	R^2^ adj. = 0.31; β = 0.04; *p* = 0.44	R^2^ adj. = 0.37; β = 0.03; *p* = 0.39
Risk of hyperglycemia	R^2^ adj. = 0.44; β = 0.06; *p* = 0.72	R^2^ = 0.66; β = 0.07; *p* = 0.68	R^2^ adj. = 0.52; β = 0.04; *p* = 0.62	R^2^ adj. = 0.62; β = 0.04; *p* = 0.68	R^2^ adj. = 0.56; β = 0.04; *p* = 0.42	R^2^ adj. = 0.58; β = 0.05; *p* = 0.52	R^2^ adj. = 0.03; β = −0.34; *p* = 0.05*	R^2^ adj. = 0.07; β = −0.37; *p* = 0.04*	R^2^ adj. = 0.04; β = −0.24; *p* = 0.02*
Total BAPAD-1 score	R^2^adj. = 0.68; β = 0.08; *p* = 0.54	R^2^ = 0.64; β = 0.05; *p* = 0.65	R^2^ adj. = 0.67; β = 0.07; *p* = 0.52	R^2^ adj. = 0.01; β = −0.12; *p* = 0.05*	R^2^ adj. = 0.02; β = −0.14; *p* = 0.04*	R^2^ adj. = 0.04; β = −0.12; *p* = 0.01*	R^2^ adj. = 0.07; β = −0.53; *p* = 0.002*	R^2^ adj. = 0.06; β = −0.55; *p* = 0.003*	R^2^ adj. = 0.03; β = −0.19; *p* = 0.01*

*Denotes a significant relationship. BMI, body mass index; HbA1c, glycated hemoglobin; PA, physical activity; MVPA, moderate to vigorous physical activity; VPA, vigorous physical activity; BAPAD-1, Barriers to Physical Activity in Type 1 Diabetes; h, hour; min, minute.

Our results reported a significant correlation between HbA1c (%) and VPA levels (β = −0.54, *p* = 0.02; β = −0.47, *p* = 0.03; β = −0.62, *p* = 0.01) for V0, V1, and V2, respectively. BMI percentile correlated with total screen time (β = 0.28, *p* = 0.02; β = 0.29, *p* = 0.01; and β = 0.31, *p* = 0.04 for V0, V1, and V2, respectively) and overall PA levels (β = −0.52, *p* = 0.02; β = −0.42, *p* = 0.03; β = −0.42, *p* = 0.01).

Finally, regression models for “sometimes experience PA hypoglycemia” and for “sometimes experience PA hyperglycemia” were examined (Table 2). There was a significant positive correlation between these responses of “sometimes experience PA hypoglycemia” and average time spent in MVPA per day across visits (β = 0.61, *p* = 0.001; β = 0.39, *p* = 0.001; and β = 0.21, *p* = 0.03 for V0, V1, and V2, respectively). The response “sometimes experience PA hyperglycemia” was significantly correlated with daily total screen time (β = 0.21, *p* = 0.04; β = 0.39, *p* = 0.03; and β = 0.51, *p* = 0.01 for V0, V1, and V2, respectively) as well as with the number of minutes of MVPA per day (β = 0.41, *p* = 0.003; β = 0.33, *p* = 0.06; and β = 0.31, *p* = 0.04 for V0, V1, and V2, respectively).

## Discussion

To the best of our knowledge, this 2-year follow-up study is the first to examine PA levels and their association with HbA1c and fear of hypoglycemia scores among youth with T1D. Our results indicated that engaging in more VPA is associated with lowered diabetes-specific barriers, primarily the fear of hypoglycemia and HbA1c. Considering that improving HbA1c with PA and increasing adherence to PA remains a major concern for pediatric diabetes, this study may highlight the importance of VPA in amplifying the benefits of PA and increasing PA engagement and overall PA.

Regardless of the diabetes group (children vs. adolescent), self-reported PA levels indicate that among our participants, 45% of children vs. 50% of adolescents cumulated at least 60 min per day of MVPA, as recommended by the Centers for Disease Control and Prevention, the American Heart Association, and the American Diabetes Association (Eckel et al., 2014; Colberg et al., 2016). Despite the greater importance of PA among individuals with T1D, our results confirm those of the literature that reported that most of the pediatric population (Troiano et al., 2008; Colley et al., 2011), and even more youths with pediatric diabetes (Michaud et al., 2017), do not meet PA guidelines.

Barriers to PA practices have been addressed as a main factor in understanding low PA levels among individuals with T1D. Indeed, those with higher scores for diabetes barriers have lower participation in PA, as reported by Michaud et al. (2017) regarding the pediatric population as well by Brazeau et al. (2012a) with respect to adults. Accordingly, for both children and adolescents, a higher barriers score has been reported for those who spent significantly less time in MVPA per day, and this pattern was seen across all visits. In fact, the accumulation of high MVPA scores may reduce overall barriers to PA practices; however, the “fear of hypoglycemia” was the only barrier’s item that seemed to be unchanged by high levels of MVPA, as per our results. Given that the “fear of hypoglycemia” is the main barrier to PA (Jabbour et al., 2016; Keshawarz et al., 2018), it is still currently unclear which mode of PA may favor an appropriate glycemic balance among individuals with T1D. Consistent with our results, a meta-analysis by Hasan et al. (2018) in laboratory-based settings reported that among adults with T1D, engaging in moderate exercise appears to be associated with a high risk of hypoglycemia, and high-intensity exercise may be safer because of a lower decline in blood glucose.

Consistent with the abovementioned findings, recent strategies have indicated that certain levels of exercise intensity could prevent hypoglycemia in T1D (Guelfi et al., 2005; Iscoe and Riddell, 2011). These experimental studies suggested that high-intensity exercise might attenuate hypoglycemia due to a greater increase in catecholamines and growth hormone and hence in glucose hepatic production (Bussau et al., 2007). Despite this promising alternative of reducing hypoglycemia episodes, we cannot assume that individuals with diabetes will become more active. Interestingly, a lower “fear of hypoglycemia” score with fewer “PA-hypoglycemia experiences” across visits was observed in the current study for those who had a high level of VPA, even when participants cumulated or not ≥ 60 min per day and had less screen time per day (< 2 h). Engaging in high VPA levels has been observed in children and was maintained across visits. In contrast, adolescents with diabetes accumulated more MVPA than their counterparts did. However, due to the design of the study, causality between VPA levels and scores for “fear of hypoglycemia” and “PA-hypoglycemia experience” cannot be inferred. Moreover, an increase in VPA levels does not guarantee any increases in overall PA levels in individuals with diabetes. Therefore, determining the required level of VPA for youth with diabetes to improve and explore other associated health outcomes may constitute promising targets to align with current guidelines.

Another important result derived from our study is that performing more VPA a day is associated with better HbA1c percentage in youth with T1D. In addition, the analysis revealed that a higher HbA1c percentage in youth with T1D was associated with increased screen time. No positive impact in HbA1c was observed in relation to the time spent in MVPA. According to our results, a study by Carral et al. (2013) reported that practicing intense PA lowered HbA1c in adults with T1D. Moreover, other intervention studies (Campaigne et al., 1984; Harmer et al., 2007) have shown similar results, but it is still difficult to draw clear conclusions that can be extrapolated to clinical practice since these intervention studies have been conducted in small samples of patients for a very limited time and with highly variable protocols. Regarding the screen time exposure, it is well-established that this outcome is linked to poorer glucose regulation in youth with T1D (Galler et al., 2011). According to general pediatric recommendations, our adolescents with T1D are more exposed to screens per day compared to their counterparts, e.g., children, which is in line with the general pediatric population (Matthews et al., 2008; Colley et al., 2011). Such result stressed the need for providing specific screen time guidelines for children and adolescents with T1D.

The current study has inherent limitations. The first challenge is that the present work relies on self-reported and not objectively measured indicators, despite the use of a validated questionnaire retrieved from a recent national survey. Therefore, supplementing the self-reported data with an objective PA measure (accelerometer) will be ideal to ensure accurate PA profiling. Second, many factors (e.g., insulin dose and carbohydrate consumption), which are largely involved in glycemic control and in preventing exercise-induced hypoglycemia, have not been addressed in the current work. It is possible that those who are engaged in more VPA were simply well aware and more informed about strategies to prevent hypoglycemia. Therefore, considering these elements will be an asset in supporting such studies. Finally, in the present study, it was really difficult to evaluate the progression of outcomes (e.g., PA, HbA1c) within our participants at different visits. Some of the main barriers were the study design itself and the lack of control. Besides the low number of participants and the tool used (e.g., PA questionnaire). This important topic, e.g., outcomes variation and their influences on glycemic parameters, might be possible under a controlled study in response to an education campaign or to a program promoting PA practices.

In conclusion, although meeting the current guidelines in terms of MVPA levels might potentiate the overall health parameter in T1D youth, these recommendations are still not clear with respect to what is the best advice for PA, which may favor metabolic control and reduce the occurrence of hypoglycemia episodes. Our results indicated that youth with T1D engaged in more VPA per day had fewer diabetes-specific barriers, primarily the fear of hypoglycemia with better HbA1c. Such results may highlight the importance of integrating VPA to amplify the benefits of PA and probably increase PA engagement and overall PA among pediatric diabetic patients.

## Data Availability Statement

The raw data supporting the conclusions of this article will be made available by the authors, without undue reservation.

## Ethics Statement

The studies involving human participants were reviewed and approved by the ethics committee of the Vitalité Health Network approved the project. Written informed consent to participate in this study was provided by the participants’ legal guardian/next of kin.

## Author Contributions

GJ contributed to the conception and design of the study and to the data collection, performed data analysis and interpretation, drafted the manuscript and revised, read, and approved the submitted version.

## Conflict of Interest

The authors declare that the research was conducted in the absence of any commercial or financial relationships that could be construed as a potential conflict of interest.
