# Supplementary material for: Vigorous Physical Activity Is Associated With Better Glycated Hemoglobin and Lower Fear of Hypoglycemia Scores in Youth With Type 1 Diabetes: A 2-Year Follow-Up Study
Source: Front Physiol. 2020 Oct 23;11:548417. doi: 10.3389/fphys.2020.548417 (PMC7645069; doi:10.3389/fphys.2020.548417)
Supplement: Supplementary file 1 [file Data_Sheet_1.PDF]

QUESTIONNAIRE – TYPE 1 DIABETES

Hello,

First off, we would like to thank you for taking the time to fill out this questionnaire, which will be used to document the lifestyles of children and adolescents with type 1 diabetes in you region.

We ask you to take your time to answer all of the questions to the best of your ability.

This is not a test; there are no correct or incorrect answers. We ask that your answers reflect your current situation.

This questionnaire is completely confidential. None of your family or friends will have access to your answers. Even our team of researchers that will analyse the questionnaire won't have access to your personal information. Only the head researcher will have access to the link between your name and questionnaire number.

This questionnaire is comprised of 2 sections. Section 1 is to be answered by the child or adolescent. An adult may assist them if they are under the age of 12 years old. Section 2 is to be answered by one of the child or adolescent's parents.

QUESTIONNAIRE – TYPE 1 DIABETES

SECTION 1 – CHILD OR ADOLESCENT

1. City or Town of residence

2. Postal Code

3. Age

years old

4. Gender (Circle one)

Female Male

5. Education (current grade or last completed)

6. Height

centimeters

or

feet inches

7. Weight

kilograms (kg)

or

pounds (lbs)

8. At what age were you diagnosed with Type 1 Diabetes?

years, and month(s) old

9. Do you use an insulin pump? (Circle one)

Yes No

QUESTIONNAIRE – TYPE 1 DIABETES

10. Please check all methods of transport that you use to go to and from school in a typical week.

- [ ] School bus
- [ ] Walk
- [ ] Bicycle
- [ ] Car
- [ ] Other (Please specify): ____

11. Which of the following statements correspond most to your current situation?

	Yes	No
My gym teacher encourages me a lot to participate in sports.		
My gym teacher limits my participation in certain activities.		
My parents do not permit me to participate in my gym class at school.		
My parents encourage me to always participate in my gym class at school.		
My doctor limits my participation in certain activities in my gym class at school.		
My doctor always encourages me to participate in my gym class at school.		
During my participation in physical activity, I sometimes have hypoglycaemic episodes.		
During my participation in physical activity, I sometimes have hyperglycaemic episodes.		

QUESTIONNAIRE – TYPE 1 DIABETES

12. Indicate the likelihood that each of these items would keep you from practicing regular physical activity during the next 6 months.

	Extremely unlikely (1)	Very unlikely (2)	Slightly unlikely (3)	Neither likely nor unlikely (4)	Slightly likely (5)	Very likely (6)	Extremely likely (7)
The loss of control over your diabetes							
The risk of hypoglycaemia							
The fear of hurting yourself							
A low fitness level							
The fact that you have diabetes							
The risk of hyperglycaemia							
Your actual physical health status excluding your diabetes							
Weather conditions							
The location of a gym							
Your school schedule							

13. In the past month, have any of the following things been a problem for you?

	Never (0)	Almost never (1)	Sometimes (2)	Often (3)	Almost always (4)
I have difficulty to walk further than my street corner.					
I have difficulties to run.					
I have difficulties practicing physical activities or doing exercise.					
I have difficulties lifting big objects.					
I have difficulties helping out around the house.					
I am in pain.					
I am lacking energy.					

14. On average, how many hours of sleep do you get per night?

hours

QUESTIONNAIRE – TYPE 1 DIABETES

SECTION 1 – Children (12 years and older)

1. In the past 3 months, which of the following activities have you done?

	How many times have you done this activity in the past 3 months?	Approximately how much time do you spend doing this activity each time
Walk for exercise		
Gardening or outdoor work		
Swimming		
Bicycling		
Dancing		
Exercising at home		
Ice Hockey		
Ice skating		
Rollerblading		
Jogging or running		
Golf		
Exercise classes or aerobics		
Skiing or snowboarding		
Bowling		
Baseball or softball		
Tennis		
Weight & resistance training		
Fishing		
Volleyball		
Basketball		
Soccer		
Other : ____________________________		
Other : ____________________________		
Other : ____________________________		

QUESTIONNAIRE – TYPE 1 DIABETES

2. In the past 3 months, on an average week, how many hours do you usually spend walking to get to work, school or to do errands?

- [ ] I don’t do any
- [ ] Less than an hour
- [ ] Between 1 and 5 hours
- [ ] Between 6 and 10 hours
- [ ] Between 11 and 20 hours
- [ ] More than 20 hours

3. In the past 3 months, on an average week, how many hours do you usually spend bicycling to get to work, school or to do errands?

- [ ] I don’t do any
- [ ] Less than an hour
- [ ] Between 1 and 5 hours
- [ ] Between 6 and 10 hours
- [ ] Between 11 and 20 hours
- [ ] More than 20 hours

4. If you think of the past 3 months, which of the following statements best describes your daily activities or work habits?

- [ ] Normally sitting down in the day, without walking much
- [ ] Often standing or walking throughout the day, without having to lift objects.
- [ ] Normally have to lift or move light objects or often going up stairs or inclined surfaces.
- [ ] Need to do manual labour / move heavy objects.

5. In the past 3 months, on an average week, how many hours do you usually spend

using a computer, including the Internet, to play games, send or receive emails, or online chatting? (Excluding time spent at school or work)

- [ ] None
- [ ] Less than an hour
- [ ] Between 1 and 2 hours
- [ ] Between 3 and 5 hours
- [ ] Between 6 and 10 hours
- [ ] Between 11 and 14 hours
- [ ] Between 15 and 20 hours
- [ ] More than 20 hours
- [ ] I don't know

6. In the past 3 months, on an average week, how many hours do you usually spend: playing video games, including XBOX, Nintendo, and PlayStation?

- [ ] None
- [ ] Less than an hour
- [ ] Between 1 and 2 hours
- [ ] Between 3 and 5 hours
- [ ] Between 6 and 10 hours
- [ ] Between 11 and 14 hours
- [ ] Between 15 and 20 hours
- [ ] More than 20 hours
- [ ] I don't know

7. In the past 3 months, during an average week, how many hours do you usually

spend: watching television, DVD's or videos?

- ☐ None
- ☐ Less than an hour
- ☐ Between 1 and 2 hours
- ☐ Between 3 and 5 hours
- ☐ Between 6 and 10 hours
- ☐ Between 11 and 14 hours
- ☐ Between 15 and 20 hours
- ☐ More than 20 hours
- ☐ I don't know

8. In the past 3 months, during an average week, how many hours do you usually spend: reading, not including the time spent doing this at school or work? Include time spent reading books, magazines, newspapers and homework for school.

- ☐ None
- ☐ Less than an hour
- ☐ Between 1 and 2 hours
- ☐ Between 3 and 5 hours
- ☐ Between 6 and 10 hours
- ☐ Between 11 and 14 hours
- ☐ Between 15 and 20 hours
- ☐ More than 20 hours
- ☐ I don't know

9. The questionnaire was filled in by: ______________________________

SECTION 1 – Children (under 12 years old)

QUESTIONNAIRE – TYPE 1 DIABETES

This page is to be filled uniquely for children under 12 years old.

Physical activity is an activity that increases heart rate and causes shortness of breath. We can do physical activity by practicing a sport, playing with friends or by walking to school.

Here are a few examples of physical activities:

- Running,
- Walking fast,
- Dancing or swimming,
- Rollerblading,
- Skateboarding,
- Bicycling,
- Playing soccer, basketball or football.

For the following 2 questions, add all of the time spent doing physical activity each day.

1. In the past 7 days, how many days were you physically active for at least 60 minutes?

☐ Never (0 days)

☐ 1 day

☐ 2 to 3 days

☐ 4 days or more

2. In an average week, how many days are you physically active for at least 60 minutes?

☐ Never (0 days)

☐ 1 day

☐ 2 to 3 days

☐ 4 days or more

3. Approximately how many hours per week do you normally spend doing physical

activity (that causes shortness of breath or makes you feel warmer than usual) in your free time at school (for example at lunch time)?

- [ ] None
- [ ] Less than an two hours per week
- [ ] Between 2 and 3 hours per week
- [ ] Between 4 and 6 hours per week
- [ ] 7 hours or more per week

4. Approximately how many hours per week do you normally spend doing physical activity (that causes shortness of breath or makes you feel warmer than usual) during class hours at school?

- [ ] None
- [ ] Less than 2 hours per week
- [ ] Between 2 and 3 hours per week
- [ ] Between 4 and 6 hours per week
- [ ] 7 hours or more per week

5. How would you describe your participation in sporting activities during school hours (for example: physical education / gym class)?

- [ ] I am active the entire class
- [ ] I sometimes take breaks
- [ ] I often take breaks

6. Approximately how many hours per week do you normally spend doing physical

activity (that causes shortness of breath or makes you feel warmer than usual): outside of school when you participate in classes, sports leagues or teams?

- [ ] None
- [ ] Less than 2 hours per week
- [ ] Between 2 and 3 hours per week
- [ ] Between 4 and 6 hours per week
- [ ] 7 hours or more per week

7. Approximately how many hours per week do you normally spend doing physical activity (that causes shortness of breath or makes you feel warmer than usual): outside of school when you participate in non-organized activities, either alone or with friends?

- [ ] None
- [ ] Less than 2 hours per week
- [ ] Between 2 and 3 hours per week
- [ ] Between 4 and 6 hours per week
- [ ] 7 hours or more per week

8. On average, how many hours do you spend per day watching television or movies or playing video games (XBOX, Nintendo, PlayStation, etc.)?

- [ ] I don’t watch television, movies or play video games
- [ ] Less than an hour per day
- [ ] Between 1 and 2 hours per day
- [ ] Between 3 and 4 hours per day
- [ ] Between 5 and 6 hours per day
- [ ] More than 7 hours per day

9. On average, how many hours do you spend per day using the computer (doing

homework, playing games, sending or receiving messages, chatting, or navigating on the internet, etc.)?

- ☐ I don't use computers
- ☐ Less than an hour per day
- ☐ Between 1 and 2 hours per day
- ☐ Between 3 and 4 hours per day
- ☐ Between 5 and 6 hours per day
- ☐ More than 7 hours per day

10. This questionnaire was filled out by:

- ☐ The child
- ☐ The child with assistance of a parent
- ☐ The parent

QUESTIONNAIRE – TYPE 1 DIABETES

This questionnaire can be completed by one of the 2 parents (mother, father or legal guardian).

In the event where the child is under joint custody, please consider only your family environment and not that of the other parent's.

In this questionnaire, several questions will refer to "your child". In your answers, we ask you to refer to your child with type 1 diabetes.

1. In the past 3 months, which of the following activities have you done?

- ☐ Walking for exercise
- ☐ Gardening or outdoor work
- ☐ Swimming
- ☐ Bicycling
- ☐ Dance
- ☐ Exercising at home
- ☐ Ice hockey
- ☐ Skating on ice
- ☐ Roller blading
- ☐ Jogging or running
- ☐ Golf
- ☐ Exercise classes or aerobics
- ☐ Skiing or snowboarding
- ☐ Bowling
- ☐ Baseball or softball
- ☐ Tennis
- ☐ Weight & resistance training
- ☐ Fishing
- ☐ Volleyball
- ☐ Basket ball
- ☐ Soccer
- ☐ Other: ____________________________

2. In the past 3 months, on an average week, how many hours do you usually spend walking to get to work, school or to do errands?

- ☐ I don't do any
- ☐ Less than an hour
- ☐ Between 1 and 5 hours
- ☐ Between 6 and 10 hours
- ☐ Between 11 and 20 hours
- ☐ More than 20 hours

3. In the past 3 months, on an average week, how many hours do you usually spend

bicycling to get to work, school or to do errands?

- [ ] I don't do any
- [ ] Less than an hour
- [ ] Between 1 and 5 hours
- [ ] Between 6 and 10 hours
- [ ] Between 11 and 20 hours
- [ ] More than 20 hours

4. If you think of the past 3 months, which of the following statements best describes your daily activities or work habits?

- [ ] Normally sitting down in the day, without walking much
- [ ] Often standing or walking throughout the day, without having to lift objects.
- [ ] Normally have to lift or move light objects or often going up stairs or inclined surfaces.
- [ ] Need to do manual labour / move heavy objects.

5. Normally, do you do physical activities with your child in your spare time?

- [ ] No, never
- [ ] Yes, 1 to 2 times per week
- [ ] Yes, 3 to 4 times per week
- [ ] Yes, 5 times or more per week

6. Are there sports and recreation facilities within proximity to your home?

- [ ] No
- [ ] Yes

7. Does your child belong to one or more sports related organizations at school or elsewhere?

QUESTIONNAIRE – TYPE 1 DIABETES

☐ No (If so, please proceed to question 11)

☐ Yes (If so, please proceed to question 8)

8. How many times per week does your child practice these sports (including practices and games)?

____

9. Are you implicated in your child's sporting organization (Example: coach)?

☐ No, never

☐ Yes, sometimes

☐ Yes, often

10. Are you present during your child's sports activities (Example: attend games)?

☐ No, never

☐ Yes, sometimes

☐ Yes, often

11. Do you provide transportation for your child to be able to practice activities?

☐ No, never

☐ Yes, sometimes

☐ Yes, often

12. To your knowledge, does your child participate in physical activities in his spare time at school (During recess, lunch hours, etc.)?

- [ ] No, never
- [ ] Yes, 1 to 2 times per week
- [ ] Yes, 3 to 4 times per week
- [ ] Yes, 5 times or more per week

13. To your knowledge, does your child participate in physical activities in his spare time at home or outside?

- [ ] No, never
- [ ] Yes, 1 to 2 times per week
- [ ] Yes, 3 to 4 times per week
- [ ] Yes, 5 times or more per week

14. Did your child watch television yesterday?

- [ ] No
- [ ] Yes, for less than 30 minutes
- [ ] Yes, between 30 minutes and one hour
- [ ] Yes, for more than one hour

15. Did your child use a computer or play video games yesterday?

- [ ] No
- [ ] Yes, for less than 30 minutes
- [ ] Yes, between 30 minutes and one hour
- [ ] Yes, for more than one hour

16. How do you consider your child to be in comparison to other children of the same age?

QUESTIONNAIRE – TYPE 1 DIABETES

☐ Never active

☐ Almost never active

☐ Sometimes active

☐ Often active

☐ Almost always active

17. In the past month, to what extent have the following things been a problem for your child?

	Never	Almost never	Sometimes	Often	Almost always
Has difficulty walking further than the street corner.					
Has difficulties running.					
Has difficulties practicing physical activities or doing sports.					
Has difficulty lifting heavy objects.					
Has difficulties taking a bath or shower without assistance.					
Has difficulty helping out with chores around the house.					
Is in pain.					
Is lacking energy.					

18. In the past month, to what extent have these things been a problem for your child regarding his emotional state?

	Never	Almost never	Sometimes	Often	Almost always
Is scared.					
Feels sad or depressed.					
Feels anger					
Has trouble sleeping					
Worries about what is going to happen to him.					

19. In the past month, to what extent have these things been a problem for your child regarding his social life?

QUESTIONNAIRE – TYPE 1 DIABETES

	Never	Almost never	Sometimes	Often	Almost always
Has trouble getting along with other children.					
Other children don't want to be his friend.					
Other children bother your child.					
Is not able to do things that other children their age are able to do.					
Isn't able to keep up when playing with other children.					

20. In the past month, to what extent have these things been a problem for your child regarding his academic performance?

	Never	Almost never	Sometimes	Often	Almost always
Has trouble listening in class.					
Forgets things					
Has difficulty doing all of their homework.					
Misses school because they don't feel well.					
Misses school to go to the doctor or the hospital.					

21. How old are you?

☐ years old

22. Are you the:

☐ Mother of the child

☐ Father of the child

☐ Other (Legal guardian)

THANK YOU!
